# The Impact of the COVID-19 Pandemic on the Mental Health of Urban Residents—Evidence from China

**DOI:** 10.3390/ijerph192316190

**Published:** 2022-12-03

**Authors:** Ying Cui, Yue Han

**Affiliations:** School of Economics, Capital University of Economics and Business, Beijing 100070, China

**Keywords:** COVID-19 pandemic, mental health, instrumental variable, mechanism analysis, long-term effect

## Abstract

Based on a nationwide micro-survey in China from 2018 to 2021, this paper empirically estimates the causal impact of the COVID-19 pandemic on the mental health of Chinese residents, by exploiting the distribution of the outflow population from Wuhan as an instrumental variable (IV). Our findings suggest that for every 10% increase in the cumulative confirmed cases, the number of mentally unhealthy days reported by urban residents in the past 30 days will increase by 2.19, an increase of 46.90% compared with the mean value. The impact is more significant among females, people aged 30 or above, and private-sector employees. Further evidence highlights the negative impact of the COVID-19 pandemic on residents’ expectations of future income and confidence in macroeconomic development, both of which we interpret as mechanisms related to economic concerns. In addition, application of the multi-period difference-in-differences (DID) strategy revealed that the negative impact still exists two years post-pandemic, but it has been dramatically alleviated since the initial stage.

## 1. Introduction

The COVID-19 pandemic was declared a Public Health Emergency of International Concern by the World Health Organization (WHO) on 30 January 2020. As of 30 September 2022, over 614 million confirmed cases of COVID-19 had been reported globally [1]. Over 250 thousand people from across all areas of China had been infected [2], more than 47 times the number of people infected with SARS in 2003 [3]. In the face of the sudden outbreak, the Chinese government imposed a series of strict measures, including, notably, the suspension of public gatherings and the lockdown of cities with severe infections. Owing to active prevention and control measures in China, the pandemic was effectively controlled at the initial stage of the outbreak. Even so, the pandemic still had a severe impact on people’s daily life, as was reported by 79.2% of respondents in a survey involving nearly 3000 urban residents [4]. COVID-19 not only affects people’s physical health, such as damage to the respiratory system and immune system, but also seriously affects people’s mental health. Theoretically, psychologist Gerald Caplan believes that each of us is trying to maintain a stable state of mind to achieve a balance between ourselves and the environment. The occurrence of stressful events may make it difficult for individuals to deal with, and the balance will be broken. This may lead to disorientation or even disorder of thinking and behavior, which means that individuals can fall into psychological crises [5]. Empirically, a survey conducted by the Chinese Academy of Social Sciences showed that at the beginning of the outbreak, people’s uncertainty about the pandemic triggered an intense emotional response, and people were in a state of stress during the one-month long survey period [6].

According to the definition put forward by WHO, “health” refers to physical and mental well-being, and a good state of social adaptation. In other words, mental health is an essential part of overall health. Formally speaking, “mental health” symbolizes a sustained mental state, in which the individual is full of vitality, has positive internal experiences, and can adapt well to the society [7]. Mental health is very important for personal growth and development, and also contributes to the spiritual civilization of the whole society. The Chinese government has paid greater attention to the mental health of residents. For example, in July 2019, the State Council issued a guideline to implement the “Healthy China” initiative, and launched a series of mental health promotion activities. Following the outbreak of the pandemic, the National Health Commission immediately issued guidelines for “Emergency Psychological Crisis Intervention.” Mental health institutions actively respond to the guidelines and are committed to providing assistance to those who may suffer from psychological distress.

In this context, we attempted to identify the causal impact of the COVID-19 pandemic on the mental health of Chinese residents. Specifically, using the National Urban Life Quality Survey data, we constructed a 2SLS model and exploited the distribution of the outflow population from Wuhan as an instrumental variable (IV). We also attempted to examine the heterogeneous effects from several aspects and the influential mechanisms from the perspective of macroeconomic expectations. Moreover, we further analyzed how the impact of the pandemic on mental health changes over time.

The remainder of the paper is organized as follows: Section 2 provides a review of related literature; Section 3 introduces the data, variables, and the econometric model; Section 4 reports the results, including the results of baseline regression, robustness test, heterogeneity analysis, and mechanism analysis; Section 5 provides an extensive analysis of long-term effects. In Section 6, we discuss the results of this paper and give an outlook to future research. In Section 7, we conclude.

## 2. Literature Review

A growing body of studies has focused on the mental health of residents during the COVID-19 pandemic. Using varied methods, they overwhelmingly concluded that there was a significant negative impact on mental health. For example, Zhang et al. [8] conducted a survey in Brazil and found that 52% of respondents experienced mild or moderate distress. According to a survey conducted in Italy by Rossi et al. [9], 20.8% of respondents suffered from anxiety and 17.3% suffered from depression. Ueda et al. [10] conducted a survey in Japan and found that 11.0% and 17.4% of respondents suffered from moderate and severe anxiety and depression, respectively. Some Chinese studies also showed that the pandemic had a strong impact on the mental health of residents. Chi et al. [11] surveyed college students and identified a 30.8% prevalence of post-traumatic stress symptoms one month after the outbreak, almost as high as the level (31.2%) after the SARS pandemic [12]. Wang et al. [13] conducted a survey involving residents from 194 cities in China. Of those surveyed, 53.8% thought the pandemic had a moderate or even severe impact on their mental health. Qiu et al. [14] conducted a survey covering more than 50,000 residents across China and found that 35% of respondents experienced psychological distress during the pandemic. In addition, the existing literature paid attention to the heterogeneous effects of the COVID-19 pandemic on different groups. They found that the pandemic had a significantly stronger impact on females [9,13,14,15,16], the unemployed [10,17], and those in the private sector [18]. Medical workers suffered from more psychological problems [19,20], as they treated front-line patients and faced a high risk of infection. There was no consensus on age heterogeneity. Some studies found that young adults were more affected [9,19,21], and others found that the elderly were more vulnerable [17,22]. Although the association between the COVID-19 pandemic and mental health has been extensively researched, there appears a lack of empirical evidence on the causality. Therefore, our paper found a relatively exogenous instrumental variable (IV) to identify the causal impact of the pandemic on mental health.

Furthermore, the existing literature has focused on the factors affecting resident mental health during the pandemic. Four main influencing factors have been identified: (1) As a new paradigm for business communication, social media applications promoted enterprises to penetrate markets that were not physically accessible [23]. However, exposure to news and information about COVID-19 on social media could elevate mental health problems [24,25,26]. (2) Social distancing policies [27,28,29,30] and livelihood insecurities [24] negatively impacted mental health. (3) Receiving social support, including material and spiritual help, could alleviate the psychological problems [26,31]. (4) The mental health of residents is less affected in a region with sufficient medical resources, efficient public health systems, and effective pandemic prevention and control measures [14,32]. These valuable conclusions unfortunately provide little evidence on the influential mechanisms related to economic concerns. To make up for this deficiency, our paper examined the mechanisms from two aspects, namely, residents’ expectations of future income and confidence in macroeconomic development. We found that the COVID-19 pandemic may influence residents’ mental health indirectly through its impact on their expectations of future income and confidence in macroeconomic development.

Additionally, all of the above literature only analyzed residents’ mental health status in the initial stage of the pandemic and provided little evidence on the long-term effects. Contrastingly, this paper explored the long-term impact of the pandemic on mental health by employing the multi-period DID strategy. We observed that the negative impact still exists two years post-pandemic, but it has been dramatically alleviated since the initial stage.

## 3. Materials and Methods

### 3.1. Data

We used the data from the National Urban Life Quality Survey, a large-scale, nationally representative study on Chinese citizens conducted by the Capital University of Economics and Business. The survey was conducted on the Sojump platform, a professional platform for online survey, evaluation, and voting. The survey covered 35 major cities in China, including 4 municipalities directly under the Central Government, 26 provincial capitals, and 5 sub-provincial cities (Shenzhen, Qingdao, Dalian, Xiamen, and Ningbo). The nationwide baseline survey was launched in 2011, and the follow-up surveys were conducted annually. The survey results were announced at the annual China Economic Growth and Business Cycle Forum, and a series of Blue Books of Urban Life Quality based on the results have been published. In this paper, we focused on the tenth wave conducted from 31 March to 13 April 2020. The survey involved 10,364 participants and provided comprehensive information on demographics, consumer confidence, education quality, health status, medical services, and the COVID-19 pandemic-related information. Furthermore, we used four waves of survey data (from 2018 to 2021) for extensive analysis, which involved 42,478 participants in total.

In addition, the pandemic-related data of each city came from the website of the Chinese Center for Disease Control and Prevention. What we focused on was the number of cumulative confirmed cases daily updated in 35 major cities. The distribution data of the outflow population from Wuhan came from Baidu Migration, a migration map provided by Baidu company. Considering the data availability, we collected data on the outflow population from Wuhan to various provinces between 10 January and 15 March 2020. This period was right before the National Urban Life Quality Survey was done.

### 3.2. Variables

#### 3.2.1. Dependent Variable

The dependent variable was the mental health of residents, measured by the number of days that the respondents had been anxious, depressed, or emotionally out of control (mentally unhealthy) in the past 30 days. Whilst self-assessed health is somewhat subjective, the existing research has proved that it can effectively reflect the respondent’s self-perceived health status [33]. In our sample, the average number of mentally unhealthy days was 4.67. Those respondents whose family members were diagnosed or isolated due to close contact, accounting for 3.52% of our sample, reported more mentally unhealthy days. In this sub-sample, the average number of mentally unhealthy days was 5.80.

#### 3.2.2. Independent Variable

The core independent variable was the severity of the pandemic, measured by the number of cumulative confirmed cases in each city. In view of the varied submission dates, we focused on the cumulative confirmed cases in the respondent’s resident city up to his/her questionnaire submission. As a robustness test, we also presented results where the cases were confirmed in the 30 days prior to questionnaire submission, so that its statistical period coincided with that of mental health. Additionally, the confirmed cases in our statistics did not include imported cases. During the survey, these cases only appeared in a few cities and were directly quarantined, thereby having little impact on the mental health of local population. Even so, as a robustness test, we also presented results where imported cases were included in confirmed cases.

#### 3.2.3. Instrumental Variable

**Figure 1:**
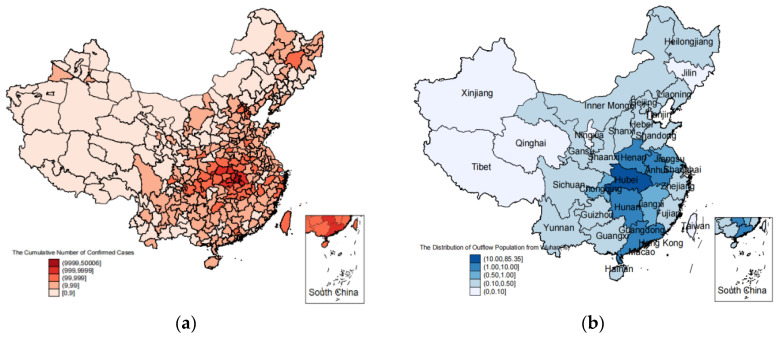
The distribution of the pandemic and the outflow population from Wuhan. (**a**) The distribution of the pandemic (as of 31 March 2020). (**b**) The distribution of the outflow population from Wuhan (10 January to 15 March 2020). Data source: Chinese Center for Disease Control and Prevention (https://www.chinacdc.cn/ (accessed on 1 August 2020)), Baidu Migration (https://qianxi.baidu.com/#/2020chunyun (accessed on 1 August 2020)). Note: Map (**a**) was drawn according to the number of cumulative confirmed cases in each city as of 31 March 2020. The darker the color, the severer the pandemic. Map (**b**) was drawn according to the percentage of the outflow population from Wuhan to each province in the total outflow population between 10 January and 15 March 2020, with a darker color indicating more outflow population. The color characteristics of the two maps are very similar, which intuitively confirms the correlation between the pandemic severity and the outflow population from Wuhan.

The mental health of residents may be affected by unobserved factors, such as commercial operations, and private and public gatherings in the city, which may relate to pandemic severity, resulting in serious endogeneity in the OLS model. Therefore, we used IV estimation to identify the real causal effects. We used the distribution of the outflow population from Wuhan as an instrumental variable for the cumulative confirmed cases in the city. It was measured by the percentage of the outflow population from Wuhan to each province in the total outflow population between 10 January and 15 March 2020. This variable meets two basic requirements for being an instrumental variable. Firstly, referring to Fang et al. [34], the outflow population from Wuhan and other cities in Hubei has a significant impact on the newly confirmed cases in the destination cities. In Figure 1, the color characteristics of the two maps are very similar, which intuitively confirms the correlation between the pandemic severity and the outflow population from Wuhan. Secondly, the outflow population from Wuhan does not correlate with the unobserved factors affecting mental health. It can only affect the mental health of residents through its impact on pandemic severity in the destination cities. Due to data limitations, the pandemic severity was measured at the city level, and the outflow population was measured at the province level. To make up for this deficiency, we introduced three city characteristics as controls into the model, which can effectively solve the endogeneity problem at the city level.

#### 3.2.4. Covariates

To minimize the estimation bias caused by omitted variables, we introduced five individual characteristics that may be associated with mental health: age, gender, years of education, monthly disposable income, and daily working hours.

Age: If the respondent is aged under 20, it takes the value of 20; if the respondent is in the age bracket of 20–29, it takes the value of 25; if the respondent is in the age bracket of 30–39, it takes the value of 35; if the respondent is in the age bracket of 40–49, it takes the value of 45; if the respondent is in the age bracket of 50–59, it takes the value of 55; if the respondent is aged 60 or above, it takes the value of 60. Different age groups face different life challenges, and their physical fitness is also quite different, which may have a great impact on their mental health.

Gender: The value of 1 indicates males, and the value of 0 indicates females. Generally speaking, females are more psychologically sensitive and more vulnerable to the negative influences from the outside world.

Years of education: If the respondent is uneducated, it takes the value of 0; if the respondent is a primary school graduate, it takes the value of 6; if the respondent is in secondary school or has graduated, it takes the value of 9; if the respondent is in college or has already had a Bachelor’s degree, it takes the value of 14; if the respondent is pursuing a Master’s degree or has already had one, it takes the value of 17. People with poor educational backgrounds face greater challenges for survival, and their mental health may be worse. However, people who are well-educated tend to have higher self-awareness of their health and may be more affected by the external environment.

Monthly disposable income: If the respondent’s income is less than RMB 3000, it takes the value of 3000; if the respondent’s income is in the bracket of RMB 3000–5000, RMB 5000–10,000, RMB 10,000–15,000, or RMB 15,000–20,000, it takes the values of 4000, 7500, 12,500, or 17,500, respectively; if the respondent’s income is more than RMB 20,000, it takes the value of 20,000. Those respondents with high income hardly need to worry about uncertainty risks. They will be more optimistic about the future, and their mental health will be improved.

Daily working hours: If the respondent works no more than 4 h a day, it takes the value of 4; if the respondent works 4–8 h a day, it takes the value of 6; if the respondent works 8–10 h a day, it takes the value of 9; if the respondent works more than 10 h a day, it takes the value of 10. The different working hours mean that individuals are under different physical and psychological pressures, which may lead to different mental health states.

In addition, we also introduced three city characteristics: GDP per capita, population density, and “lockdown” policies. These factors may influence the severity of the pandemic [35,36] and the mental health of residents. Among them, “lockdown” indicates different levels of prevention and control measures across cities. In reference to the classification criteria applied by Fang et al. [34], if the city is under complete lockdown (all public transport and private vehicles are banned, all residential buildings are locked down, and residents are not allowed to leave the city), it takes the value of 3; if the city is under partial lockdown (majority of the public transport is temporarily shut down, checkpoints are set up to control the inflow population, and each community is under surveillance), it takes the value of 2; if checkpoints and quarantine zones are set up in the city and public transport maintains normal operation, it takes the value of 1; otherwise, it takes the value of 0.

#### 3.2.5. Mechanism Variables

We explored the mechanisms from two aspects, namely, residents’ expectations of future income and confidence in macroeconomic development. We created an ordinal variable with values from 1 to 5 to measure residents’ expectations of future income. If the respondent thinks his/her future income will be much worse than the current income, it takes the value of 1. If the respondent thinks the future income will be much better than the current income, it takes the value of 5. Similarly, we created an ordinal variable with values from 1 to 5 to measure residents’ confidence in macroeconomic development. The value of 1 indicates no confidence at all, and the value of 5 indicates complete confidence.

#### 3.2.6. Descriptive Statistics

Table 1 shows the descriptive statistics of the main variables. To perform a preliminary assessment of data quality, we compared our sample with that of China Family Panel Studies (CFPS) in 2018. The CFPS is a nationally representative study that collects data from 25 provinces/municipalities/autonomous regions in China using the implicit stratified probability-proportional-to-size sampling (PPS) method. We found that the gender distribution of the Urban Life Quality Survey closely resembles that of the CFPS. The respondents in the Urban Life Quality Survey are younger and have a higher level of education, which can be explained by the fact that the survey is conducted online and most cities involved are municipalities or provincial capitals. As municipalities and provincial capitals are more affected by the COVID-19 pandemic, focusing on residents in these cities can help us better understand the impact of the pandemic on mental health. Overall, we believe our sample is a reasonable representation of Chinese citizens.

### 3.3. Econometric Model

Our benchmark OLS model is shown in Equation (1).

(1) $$Y_{ijk}=\lambda_{0}+\lambda_{1}\ln(Covid_{ijk})+\lambda_{2}X_{i}+\lambda_{3}Z_{j}+\theta_{k}+\sigma_{ijk}$$

where *Y_ijk_* indicates the number of mentally unhealthy days reported by respondent *i* who lives in city *j* of region *k*. *Covid_ijk_* indicates the cumulative confirmed cases in city *j* of region *k* up to respondent *i*’s questionnaire submission. As the submission date varied among individuals, although the number of cumulative confirmed cases is for the city level, it has variations for individuals within the same city. Given that *Covid_ijk_* is much larger in magnitude than *Y_ijk_*, we took the logarithm of *Covid_ijk_* and mark it as *ln(Covid_ijk_)*. *X_i_* and *Z_j_* contain vectors of individual and city characteristics, respectively. *θ_k_* denotes region-fixed effects for Northeastern, Eastern, Northern, Central, Southern, Northwestern and Southwestern China.

In the OLS model, the key coefficient *λ*_1_ may be biased, because the error term *σ_ijk_* may contain unobserved city-level factors related to pandemic severity and resident mental health. To address this endogeneity problem, we referred to Fang et al. [34], and constructed a two-stage least-squares (2SLS) model with the distribution of the outflow population from Wuhan as an instrumental variable. Equations (2) and (3) represent first-stage and second-stage regressions, respectively.

(2) $$\ln(Covid_{ijrk})=\alpha_{0}+\alpha_{1}Mob_{r}+\alpha_{2}X_{i}+\alpha_{3}Z_{j}+\theta_{k}+\omega_{ijrk}$$

(3) $$Y_{ijrk}=\beta_{0}+\beta_{1}\overset{\wedge }{\ln(Covid_{ijrk})}+\beta_{2}X_{i}+\beta_{3}Z_{j}+\theta_{k}+\varepsilon_{ijrk}$$

where *Mob_r_* indexes the percentage of the outflow population from Wuhan to province *r* in the total outflow population. $\overset{\wedge }{Covid_{ijrk}}$ is the fitted value of *Covid_ijrk_* in the first-stage regression. *ω_ijrk_* and *ε_ijrk_* are error terms. Other variables are defined in the same way as in Equation (1). The IV estimator of coefficient *β*_1_ captures the causal effects of *Covid_ijrk_* on *Y_ijrk_*, and is of central interest to our study.

## 4. Empirical Results

### 4.1. Baseline Regression

Table 2 reports the baseline regression results. The OLS estimates in Columns (1) and (2) indicate little or no significant correlation between pandemic severity and resident mental unhealthiness in our sample. However, the 2SLS estimates in Columns (3) and (4) are quite different from this. The coefficients of the core indicator are positively significant at the 1% level, suggesting that the pandemic had a significant adverse impact on the mental health of Chinese residents. In our preferred specification in Column (4), every 10% increase in the cumulative confirmed cases led to a 2.19 increase in mentally unhealthy days reported by residents in the previous 30 days, which signifies an increase of 46.90% compared with its mean value. The coefficient in first-stage regression is significant at the 1% level, and the F-statistic is much larger than its critical value, which can exclude the possibility of weak IVs. By comparison, we found that the OLS estimates are indeed biased, and potential endogeneity tends to underestimate the adverse effects of the pandemic on mental health. This result indicates that the OLS model omits some unobserved factors, such as commercial operations, and private and public gatherings in the city, which are positively correlated with pandemic severity and negatively correlated with mental unhealthiness.

### 4.2. Robustness Test

The above analysis confirms that the pandemic had a significant negative impact on the mental health of Chinese residents. To verify the reliability of this conclusion, we conducted a series of robustness tests. The results are shown in Table 3.

Firstly, since the estimates may be sensitive to different sample sizes among the cities, we conducted a weighted regression test based on the sample size of each city. The estimated coefficient is the same as that of baseline regression.

Secondly, another potential concern is that the estimates may be sensitive to alternative measures of IV. Considering that the lockdown policy significantly reduced the outflows from Wuhan, we took the distribution of the outflow population from Wuhan between 10 January and 22 January 2020, prior to the lockdown of Wuhan, as an alternative measure of IV. The result is quite close to that of our baseline regression. Thus, our main findings are robust to the more flexible specification of IV.

Thirdly, as the pandemic spread globally, some cities, especially those with higher levels of opening up, saw an increase in imported cases during the survey period. Thus, we used the cumulative confirmed cases, including imported cases, as an alternative measure of pandemic severity. The result is quite similar to that of our baseline regression, indicating that our main findings are robust to the alternative measures of the independent variable.

Fourthly, residents’ mental health was measured by their reported number of mentally unhealthy days in the past 30 days. Considering that mental health may have been affected by the immediate pandemic situation, we calculated confirmed cases in the 30 days prior to questionnaire submission. The estimated coefficient is slightly smaller than that of baseline regression, indicating that residents’ mental health may also have been affected, though to a lesser extent, by the pandemic situation in the early stage.

### 4.3. Heterogeneous Effects

We conducted three sets of stratified analyses to identify whether the estimated impact varied with the gender, age, and job type of respondents. The results are summarized in Table 4.

We can see from Panel A that the pandemic had a significantly stronger impact on the mental health of females than males. There are three main possible explanations for the gender differences: (1) females prefer to adopt risk coping, protective coping, and stress coping when encountering crisis, whereas males prefer informational coping [37], so females tended to show more emotional responses during the pandemic; (2) as schools and childcare centers were closed during the pandemic, females needed to take more responsibility for the care-giving of children [38,39], and they were faced with a heavier burden of housework; (3) service industries such as catering, accommodation, and tourism were hardest hit by the pandemic, in which females account for the majority and were more likely to suffer from job loss or reduced income.

We can see in Panel B that the pandemic had a severer impact on the mental health of people aged 30 or above. The middle-aged are faced with a heavier burden of family care and greater pressure on income, so they were at higher risk of psychological distress during the pandemic. Older people usually have poorer physical fitness, so they were more likely to feel anxious and depressed about the virus [40]. The disruption of parent–child contact due to social distancing policies increased the psychological problems of older people [22].

The results in Panel C show that the impact was more evident for employees in the private sector. According to “Investigation and Analysis of the Impact of the COVID-19 on Business Development” issued by China Enterprise Reform and Development Society, the pandemic had the largest impact on individual businesses, with a 67.5% effect, followed by private enterprises, with a 54.1% effect. Employees in the private sector were faced with severe challenges in livelihood, so their mental health needed more attention.

### 4.4. Influential Mechanisms

The above results demonstrate that the COVID-19 pandemic exerted a significant impact on the mental health of Chinese residents. This section further explores the potential mechanisms from the perspectives of residents’ expectations of future income and confidence in macroeconomic development. We estimate the following:

(4) $$M_{ijk}=m_{0}+m_{1}\ln(Covid_{ijk})+m_{2}X_{i}+m_{3}Z_{j}+\theta_{k}+\tau_{ijk}$$

where *M_ijk_* represents the mechanism variables, namely, expectations of future income and confidence in macroeconomic development reported by respondent *i* in city *j* of region *k*. *τ_ijk_* is an error term. Other variables are defined in the same way as in Equation (1). The coefficient *m*_1_, which captures the causal effects of *Covid_ijk_* on *M_ijk_*, is of central interest to our study.

Table 5 reports the regression results. The estimated coefficients in Columns (1) and (2) are both negatively significant at the 1% level, indicating that the pandemic has an adverse impact on residents’ expectations of future income and confidence in macroeconomic development. Furthermore, according to existing research, expectations of future income have positive effects on individual happiness [41,42,43]. The confidence in economy correlates positively with subjective well-being and correlates negatively with depression [44]. Thus, during the COVID-19 pandemic, those residents with lower expectations and confidence tend to experience severer mental unhealthiness. In a word, our results show that besides the influential mechanisms proposed in existing literature, the pandemic may indirectly influence residents’ mental health through its impact on their expectations of future income and confidence in macroeconomic development.

## 5. Extensive Analysis

A major worldwide concern is whether the pandemic will leave a long-term legacy on mental health. In this section, we used the survey data from 2018 to 2021 and adopted the multi-period DID method to analyze how the impact of the pandemic on mental health changes over time.

Firstly, we define time dummies *T_t_*. The post-pandemic periods, the years 2020 and 2021, are indexed by *T_2020_* = 1 and *T_2021_* = 1, respectively. The pre-pandemic periods, the years 2018 and 2019, are indexed by *T_2018_* = 0 and *T_2019_* = 0, respectively. Secondly, we define the treatment and control groups. In reference to the classification criteria applied by He et al. [45], we selected six cities with more than 300 cumulative confirmed cases at the time of our survey as the treatment group. The selected cities were Wuhan, Shanghai, Beijing, Chongqing, Shenzhen, and Guangzhou, and they are denoted by *D_j_ =* 1. The other 29 cities involved in the survey served as the control group and are denoted by *D_j_* = 0. The DID specification is described as follows:

(5) $$Y_{ijkt}=\gamma_{0}+\gamma_{1}D_{j}+\gamma_{2}T_{t}+\gamma_{3}D_{j}\times T_{t}+\gamma_{4}X_{i}+\gamma_{5}Z_{j}+\theta_{k}+\mu_{t}+\upsilon_{ijkt}$$

where *Y_ijkt_* represents the number of mentally unhealthy days reported by respondent *i* in city *j* of region *k* in the year *t*. *μ_t_* denotes year-fixed effects. *υ_ijkt_* is an error term. Other variables are defined in the same way as in Equation (1). The coefficient *γ_3_*, which captures the impact of the COVID-19 pandemic on mental health, is the focus of this study. In addition, we replaced the dummy variable *D_j_* with the continuous variable *Covid_ijk_*, the cumulative confirmed cases in city *j* of region *k* up till the respondent *i*’s questionnaire submission.

In Table 6, Columns (1) and (2) report estimates based on the mixed-panel data of the years 2018, 2019, and 2020. Columns (3) and (4) report estimates based on the mixed-panel data of the years 2018, 2019, and 2021. The difference between the coefficients of *D_j_* × *T_2020_* and *D_j_* × *T_2021_* demonstrates that the negative impact on mental health still existed two years post-pandemic, but it has been dramatically alleviated in comparison to the initial stage. We can draw the same conclusion from the difference between the coefficients of *Covid_ijk_* × *T_2020_* and *Covid_ijk_* × *T_2021_*. Our conclusion is consistent with the research by Cai et al. [46]—that is, by the end of 2020, the Chinese labor market had recovered to a great extent, and the mental health of employees had also significantly improved.

The premise of DID method is that the treatment and control groups have the same time trend in the absence of policy treatment. To verify this assumption, we conducted a parallel trend test. Specifically, the definitions of treatment and control groups were the same as above. We assumed that the pandemic broke out one year earlier than it actually did. As reported in Table 7, the coefficients of *D_j_* × *T_2019_* and *Covid_ijk_* × *T_2019_* are not significant, indicating that before the outbreak of the pandemic, there was no significant difference in mental health between the treatment group and the control group. Thus, we can conclude that the two groups satisfy the parallel-trend assumption.

## 6. Discussion

The COVID-19 pandemic has touched everyone across the globe, and we are becoming ever more aware of its impact. The existing literature used various indicators, such as the prevalence of anxiety and depression, to evaluate mental health during the pandemic. Additionally, they found widespread psychological distress among respondents. However, these studies did not collect real-time pandemic data, nor did they provide evidence on the causality. In this study, we collected data on cumulative confirmed cases in each city and matched them to individual mental health information observed during the survey. Thus, we could accurately quantify the deterioration of residents’ mental health caused by the rising severity of the pandemic. Our conclusion is consistent with the research by Wang et al. [13] and Qiu et al. [14]. They conducted nationwide surveys on Chinese residents and found that the pandemic had an adverse impact on mental health. What goes further than their research is that our paper identifies the causal effects. After addressing the endogeneity problem that may underestimate the adverse impact of the pandemic on mental health, our findings suggest that for every 10% increase in cumulative confirmed cases, the number of mentally unhealthy days reported by urban residents in the past 30 days will increase by 2.19.

In heterogeneity analysis, we found that females and private sector employees are more vulnerable, which is consistent with the existing research [9,13,14,15,16,18]. The results also show that the pandemic has a significant negative impact on the mental health of people aged 30 or above, but not on those aged under 30. There is no consensus on the impact of the pandemic on different age groups [9,17,19,21,22]. The reasons may come from the following aspects: (1) these studies focused on different dimensions of mental health, including anxiety, depression, post-traumatic stress symptoms, etc.; (2) these studies adopted different age standards to divide the respondents into subgroups.

In mechanism analysis, we found that the pandemic may indirectly affect residents’ mental health through its impact on their expectations of future income and confidence in macroeconomic development. Guo et al. [24] revealed that the perceived negative impact of the pandemic on livelihood predicts an increase in mental health problems. They emphasized the negative impact of the pandemic on current livelihood, while our paper highlights the negative impact on expectations and confidence in the future. Additionally, by referring to the existing literature, we can also illustrate that expectations and confidence will further affect mental health. In fact, boosting residents’ expectations and confidence can not only improve their mental health, but also be of great significance to national economic development. According to traditional macroeconomic theory, consumers’ expectations of inflation will aggravate the actual inflation. Rational consumers make consumption decisions based not only on their current income, but also on future predicted income. If consumers expect the future economy to be full of uncertainty, they will cut back on current spending and save more to avoid the impact of future emergencies. In addition, some studies have proved the positive impact of consumer confidence on consumer demand and its signaling effects on the economic situation [47,48]. Therefore, it is necessary to stabilize employment, so as to mitigate residents’ concerns about future income. It is also necessary to guide residents to correctly understand the current economic situation and enhance their confidence in economic development.

Long-term effects analysis is one of the major contributions in our paper. Compared with the data used in existing research [13,14], our data have an obvious advantage. They are from a longitudinal annual survey, which enables us to analyze the relative long-term effects. According to the survey results in 2021, the negative impact of the pandemic on residents’ mental health has been significantly alleviated compared with that in 2020. Owing to active pandemic prevention and control measures in China, the pandemic was effectively controlled within a relatively short period of time, which brought obvious benefits to public health. Additionally, the Chinese government actively promoted employment recovery to minimize the impact of the pandemic on the labor market, which can explain the improvement in the mental health of employees. For example, the government increased investment in business projects, especially those with positive employment effects.

In the context of regular pandemic prevention and control in China, population health needs continuous attention. Due to data limitations, this paper pays insufficient attention to other social and personal factors that may affect mental health during the pandemic and lacks an analysis of health behaviors. If possible, future research can be carried out on the following aspects. Firstly, the existing studies have found that severe health shocks, such as the SARS pandemic (2003) and the H1N1 pandemic (2009), may induce positive changes in health behaviors amongst survivors, thereby leading to long-term improvements in population health [49,50]. Another study on the COVID-19 pandemic found that the pandemic improves people’s willingness to pay for their health, and such an effect can only work in the short term [51]. However, this conclusion is simply based on people’s willingness to buy healthier goods. In fact, health behaviors involve many aspects, such as regular physical exercise, physical examination, and dietary habits. Whether the COVID-19 pandemic has positive long-term effects on people’s health behaviors is worthy of future research. Secondly, the existing studies have found that institutional trust is an important factor influencing individuals’ preventive behavior during extreme health shocks [52,53]. Future research can be carried out to explore whether institutional trust has an impact on individuals’ mental health during the COVID-19 pandemic. Thirdly, strict pandemic prevention and control measures can effectively curb the spread of the virus, but tend to have a negative impact on the labor market. Thus, another important topic for future research is to tradeoff the health benefits and economic costs of pandemic prevention and control measures.

## 7. Conclusions

Based on a nationwide micro-survey in China from 2018 to 2021 and the COVID-19 pandemic data released by the Chinese Center for Disease Control and Prevention, we empirically estimated the causal impact of the pandemic on the mental health of Chinese residents. We constructed a 2SLS model and exploited the distribution of the outflow population from Wuhan as an instrumental variable (IV), which effectively addresses the endogeneity problem. The results show that the pandemic had a significant negative impact on mental health, especially among females, people aged 30 or above, and private sector employees. Besides the influential mechanisms suggested by existing literature, the pandemic may indirectly influence residents’ mental health through its impact on their expectations of future income and confidence in macroeconomic development. Moreover, this paper examined the long-term effects by adopting the multi-period DID method. The results show that the impact of the pandemic on mental health still exists two years on, but it has dramatically improved since the initial outbreak.

Our results have important policy implications. Firstly, relevant supportive policies, such as credit concessions and tax breaks for enterprises, are needed to stabilize employment and reduce residents’ concerns about future income. The sectors most affected by the pandemic are private enterprises and individual businesses. Employees in these sectors are more likely to suffer from job loss or reduced income, and face severe livelihood challenges. Thus, supportive policies should be inclined to these vulnerable sectors. Secondly, the government should not only provide subsidies for the unemployed, but also introduce employment-aided policies to help them get back to work. An increase in market demand is conducive to economic growth, and market demand will only increase when people are in stable employment and are sure of a higher future income. Thirdly, mental health problems significantly increased during the pandemic. Therefore, the government should attach importance to strengthening the construction of the social psychological service system and improving the standardized management of psychological services. Mental health institutions should actively provide assistance to people who may suffer from psychological distress.

## Figures and Tables

**Table 1:** Descriptive statistics of the main variables.

Variable	Obs.	Mean	SD	Min	Max
Dependent variable					
Mentally unhealthy days	10,364	4.67	5.69	1	30
Independent variable					
Cumulative confirmed cases (100 cases)	10,364	18.67	91.07	0.07	500.06
Instrumental variable					
Distribution of the outflow population from Wuhan (%)	10,364	3.44	15.50	0.02	85.35
Covariates					
Age	10,364	32.22	10.49	20	60
Gender	10,364	0.44	0.50	0	1
Years of education	10,364	13.11	2.53	0	17
Monthly disposable income (1000 yuan)	10,364	6.13	3.94	3	20
Daily working hours	10,364	7.09	1.89	4	10
GDP per capita (10,000 yuan)	10,364	10.93	3.68	5.29	20.35
Population density (100 people/km^2^)	10,364	10.70	12.03	1.79	66.25
“Lockdown” policies	10,364	0.90	0.76	0	3
Mechanism variables					
Expectations of future income	10,364	3.78	0.92	1	5
Confidence in macroeconomic development	10,364	4.20	0.78	1	5

Note: The data of GDP per capita and population density were from the China Urban Statistical Yearbook 2020.

**Table 2:** Baseline regression.

	Mentally Unhealthy Days			
	OLS	OLS	2SLS	2SLS
	(1)	(2)	(3)	(4)
Panel A: 2SLS results				
ln(cumulative confirmed cases)	0.118 *	0.104	0.205 ***	0.219 ***
	(0.060)	(0.063)	(0.045)	(0.056)
Age	−0.004	−0.004	−0.004	−0.004
	(0.008)	(0.008)	(0.008)	(0.008)
Gender	−0.420 ***	−0.433 ***	−0.410 ***	−0.419 ***
	(0.128)	(0.127)	(0.126)	(0.125)
Years of education	0.033	0.033	0.033	0.033
	(0.024)	(0.024)	(0.024)	(0.024)
ln(monthly disposable income)	−0.375 ***	−0.369 ***	−0.391 ***	−0.394 ***
	(0.097)	(0.092)	(0.097)	(0.092)
Daily working hours	0.233 ***	0.231 ***	0.233 ***	0.232 ***
	(0.031)	(0.031)	(0.031)	(0.031)
Panel B: First-stage results				
Distribution of the outflow population from Wuhan			0.061 ***	0.054 ***
			(0.006)	(0.009)
City characteristics	Yes	Yes	Yes	Yes
Region FE	No	Yes	No	Yes
1st F-statistic			114.522	39.751
Observations	10,364	10,364	10,364	10,364

Note: *** significant at the 1% level; * significant at the 10% level. Standard errors clustered at the city level are reported in parentheses. We included three city characteristics: GDP per capita; population density; “lockdown” policies. We included six regional dummies: Northeastern; Eastern; Northern; Central; Southern; Northwestern China (Southwestern China as the reference group).

**Table 3:** Robustness tests.

	Mentally Unhealthy Days			
	Weighted 2SLS	Using the Distribution of the Outflow Population from Wuhan Prior to the Lockdown as an Instrumental Variable	Using the Cumulative Confirmed Cases Including Imported Cases as an Independent Variable	Using the Confirmed Cases in the Past 30 Days as an Independent Variable
	(1)	(2)	(3)	(4)
ln(cumulative confirmed cases)	0.219 ***	0.215 ***	0.221 ***	0.173 ***
	(0.055)	(0.056)	(0.056)	(0.045)
City characteristics	Yes	Yes	Yes	Yes
Region FE	Yes	Yes	Yes	Yes
1st F-statistic	40.366	37.743	36.497	218.265
Observations	10,364	10,364	10,364	10,364

Note: *** significant at the 1% level. Standard errors clustered at the city level are reported in parentheses. We included five individual characteristics: age; gender; years of education; monthly disposable income; daily working hours. We included three city characteristics: GDP per capita; population density; “lockdown” policies. We included six regional dummies: Northeastern; Eastern; Northern; Central; Southern; Northwestern China (Southwestern China as the reference group).

**Table 4:** Heterogeneity analysis.

	Mentally Unhealthy Days	
	(1)	(2)
Panel A: Regression of subsamples by gender		
	Females	Males
ln(cumulative confirmed cases)	0.266 ***	0.165 **
	(0.066)	(0.077)
1st F-statistic	33.372	50.649
Observations	5849	4515
Panel B: Regression of subsamples by age		
	People aged under 30	People aged 30 or above
ln(cumulative confirmed cases)	0.074	0.375 ***
	(0.119)	(0.138)
1st F-statistic	51.827	30.811
Observations	5473	4891
Panel C: Regression of subsamples by job type		
	Employees in state-owned enterprises, government agencies, and public institutions	Employees in private enterprises and individual businesses
ln(cumulative confirmed cases)	0.125	0.286 ***
	(0.115)	(0.098)
1st F-statistic	30.713	51.340
Observations	2938	3454

Note: *** significant at the 1% level; ** significant at the 5% level. Standard errors clustered at the city level are reported in parentheses. We included five individual characteristics: age; gender; years of education; monthly disposable income; daily working hours. We included three city characteristics: GDP per capita; population density; “lockdown” policies. We included six regional dummies: Northeastern; Eastern; Northern; Central; Southern; Northwestern China (Southwestern China as the reference group).

**Table 5:** Mechanism analysis.

	Expectations of Future Income	Confidence in Macroeconomic Development
	(1)	(2)
ln(cumulative confirmed cases)	−0.042 ***	−0.033 ***
	(0.010)	(0.012)
City characteristics	Yes	Yes
Region FE	Yes	Yes
1st F-statistic	39.751	39.751
Observations	10,364	10,364

Note: *** significant at the 1% level. Standard errors clustered at the city level are reported in parentheses. We included five individual characteristics: age; gender; years of education; monthly disposable income; daily working hours. We included three city characteristics: GDP per capita; population density; “lockdown” policies. We included six regional dummies: Northeastern; Eastern; Northern; Central; Southern; Northwestern China (Southwestern China as the reference group).

**Table 6:** DID regression.

	Mentally Unhealthy Days			
	(1)	(2)	(3)	(4)
*D_j_* × *T_2020_*	0.720 ***			
	(0.160)			
*Covid_ijk_* × *T_2020_*		0.144 ***		
		(0.042)		
*D_j_* × *T_2021_*			0.296 **	
			(0.137)	
*Covid_ijk_* × *T_2021_*				0.058 *
				(0.030)
City characteristics	Yes	Yes	Yes	Yes
Region FE	Yes	Yes	Yes	Yes
Year FE	Yes	Yes	Yes	Yes
Observations	32,217	32,217	32,114	32,114
*R* ^2^	0.050	0.050	0.051	0.051

Note: *** significant at the 1% level; ** significant at the 5% level; * significant at the 10% level. Standard errors clustered at the city level are reported in parentheses. We included five individual characteristics: age; gender; years of education; monthly disposable income; daily working hours. We included three city characteristics: GDP per capita; population density; “lockdown” policies. We included six regional dummies: Northeastern; Eastern; Northern; Central; Southern; Northwestern China (Southwestern China as the reference group).

**Table 7:** Parallel trend test.

	Mentally Unhealthy Days	
	(1)	(2)
*D_j_* × *T_2019_*	0.122	
	(0.158)	
*Covid_ijk_* × *T_2019_*		−0.008
		(0.030)
City characteristics	Yes	Yes
Region FE	Yes	Yes
Year FE	Yes	Yes
Observations	42,478	42,478
*R* ^2^	0.040	0.040

Note: Standard errors clustered at the city level are reported in parentheses. We included five individual characteristics: age; gender; years of education; monthly disposable income; daily working hours. We included three city characteristics: GDP per capita; population density; “lockdown” policies. We included six regional dummies: Northeastern; Eastern; Northern; Central; Southern; Northwestern China (Southwestern China as the reference group).

## Data Availability

The data can be made available upon request.
